# Ventral Hippocampal CA1 Pyramidal Neurons Encode Nociceptive Information

**DOI:** 10.1007/s12264-023-01086-x

**Published:** 2023-07-13

**Authors:** Yue Wang, Naizheng Liu, Longyu Ma, Lupeng Yue, Shuang Cui, Feng-Yu Liu, Ming Yi, You Wan

**Affiliations:** 1 Neuroscience Research Institute and Department of Neurobiology, School of Basic Medical Sciences, Peking University, Beijing, 100083 China; 2 Key Laboratory for Neuroscience, Ministry of Education/National Health Commission, Peking University, Beijing, 100083 China; 3 Co-innovation Center of Neuroregeneration, Nantong University, Nantong, 226019 China; 4 CAS Key Laboratory of Mental Health, Institute of Psychology, Chinese Academy of Sciences, Beijing, 100101 China; 5 Department of Psychology, University of Chinese Academy of Sciences, Beijing, 100101 China

**Keywords:** Ventral hippocampal CA1, Nociception, Mechanical allodynia, *In vivo* recording, Neural coding

## Abstract

As a main structure of the limbic system, the hippocampus plays a critical role in pain perception and chronicity. The ventral hippocampal CA1 (vCA1) is closely associated with negative emotions such as anxiety, stress, and fear, yet how vCA1 neurons encode nociceptive information remains unclear. Using *in vivo* electrophysiological recording, we characterized vCA1 pyramidal neuron subpopulations that exhibited inhibitory or excitatory responses to plantar stimuli and were implicated in encoding stimuli modalities in naïve rats. Functional heterogeneity of the vCA1 pyramidal neurons was further identified in neuropathic pain conditions: the proportion and magnitude of the inhibitory response neurons paralleled mechanical allodynia and contributed to the confounded encoding of innocuous and noxious stimuli, whereas the excitatory response neurons were still instrumental in the discrimination of stimulus properties. Increased theta power and theta-spike coupling in vCA1 correlated with nociceptive behaviors. Optogenetic inhibition of vCA1 pyramidal neurons induced mechanical allodynia in naïve rats, whereas chemogenetic reversal of the overall suppressed vCA1 activity had analgesic effects in rats with neuropathic pain. These results provide direct evidence for the representations of nociceptive information in vCA1.

## Introduction

The ability to detect noxious stimuli enables the engagement of appropriate protective behaviors against harmful conditions, and hence is essential for survival [1]. However, innocuous mechanical stimuli can be perceived as painful in neuropathic pain, which is termed mechanical allodynia, and seriously affect the quality of life of patients [2, 3]. Accumulating studies have unraveled the neural mechanisms of mechanical allodynia from peripheral to central levels [4–7], yet the neural coding of innocuous and noxious information under physiological and pathological conditions remains obscure.

As a major structure of the limbic system, the hippocampus plays a critical role in pain perception and chronicity [8, 9]. Acute noxious heat stimuli evoke increased theta activation and suppressed pyramidal neuron responses in the hippocampus [10]. Persistent pain inhibits hippocampal neurogenesis, synaptic plasticity, and c-Fos expression, reduces hippocampal volume, and disrupts hippocampal functional connectivity [11–13]. The hippocampus is functionally segmented along its dorsoventral axis, with the dorsal part participating in episodic memories while the ventral part contributes to affective behaviors [14, 15]. In rodents, suppressed dorsal hippocampal activity underlies cognitive deficits in chronic pain [16, 17]. By contrast, the ventral hippocampus is more involved in the perceptual and affective aspects of pain [18–20]. Furthermore, neuronal subsets in the ventral hippocampal CA1 (vCA1) respond to anxiogenic environments [21], aversive shock [22], and social presence [23]. However, direct evidence for the representation of nociceptive information in the vCA1 is lacking.

To depict the neural coding of nociceptive information in the vCA1, we examined the electrophysiological change of vCA1 neural activity in response to diverse somatosensory stimuli and genetically manipulated the activity of vCA1 pyramidal neurons in naïve rats and rats with neuropathic pain. We conclude that nociception is characterized by the suppression of a large fraction of pyramidal neurons and the activation of a small fraction, along with enhanced theta power and theta-spike synchronization in vCA1.

## Materials and Methods

### Animals

Adult male Sprague-Dawley rats (250 ± 30 g at the beginning of experiments) were provided by the Department of Laboratory Animal Sciences, Peking University Health Science Center (Beijing, China). Rats were housed in groups of 2–3 (except for rats with implanted electrodes, which were housed singly) under a 12-h dark-light cycle with *ad libitum* access to food and water. All experimental procedures were approved by the Animal Care and Use Committee of Peking University Health Science Center, according to the guidelines of the International Association for the Study of Pain.

### Spared Nerve Injury Model of Neuropathic Pain in Rats

The neuropathic pain model of spared nerve injury (SNI) was established as previously described [24]. Rats were anesthetized with 1% pentobarbital sodium (50 mg/kg, i.p.), and the left biceps femoris muscle was sectioned to expose the sciatic nerve and its trifurcation. The common peroneal nerve and the tibial nerve were tightly ligated with 5.0 silk thread and sectioned distal to the ligation with the removal of a 2–4 mm nerve segment. The sural nerve was left intact. Muscles and skin were closed in two layers. The SNI model was considered successful if a 2.0-g von Frey filament induced at least three paw withdrawal reflexes out of five repeated stimuli. In sham-operated rats, the left sciatic nerve and its trifurcation were exposed but not manipulated.

### Electrode Construction and Implantation

Custom-designed, 3D-printed electrodes consisting of 8 movable tetrodes [25] were used for chronic *in vivo* electrophysiological recordings. The tetrodes were fashioned from tungsten wires (diameter 20 µm, California Fine Wire Co.). The impedance of each tetrode was between 1 and 1.5 MΩ.

The animals were initially anesthetized with 5% vaporized isoflurane, then head-fixed on a stereotaxic apparatus (RWD, Shenzhen, China). 1–2% vaporized isoflurane was used to maintain anesthesia throughout the surgery. Two anchoring screws were tapped into the skull for electrode attachment. Another two stainless steel screws with copper wires were set over the cerebellum as reference and ground wires. After the craniotomy, tetrodes were implanted into the right vCA1 (−4.7 to −5.7 mm anteroposterior; 5.0 to 6.2 mm mediolateral from bregma; 5.0 to 7.0 mm ventral to brain surface). The electrode was secured to the skull using dental cement and protected by a 3D-printed shell for longitudinal recording. The animals were single-housed after surgery and allowed to recover for 5–7 days before further experiments.

### In vivo Electrophysiology Recording

After recovery from the surgery, rats were handled by the experimenter for at least 3 consecutive days and habituated to the recording environment before each recording session. During habituation, tetrodes were slowly lowered to screen for spikes. Once spikes were detected, the tetrodes were kept stabilized throughout the recording session and advanced ~20 µm between recording days.

The rat was allowed to move freely in a transparent plastic chamber (30 cm × 30 cm × 40 cm) with video recording (Basler, Germany). The chamber floor was a grid plate with stainless steel bars 2 mm in diameter and 8 mm between bars. Electrophysiological data were amplified by a recording head stage and acquired using a 32-channel extracellular recording system (Intan Technologies, Los Angeles, USA).

Electrophysiology recording was performed when the rat was quietly awake. Each session contained a 20-min resting phase for recording spontaneous neural activity, followed by a stimulation phase. During this phase, four types of plantar stimuli were applied to the left hind paw in the following order: brush stroking the surface from heel to toe (dynamic innocuous touch), von Frey filament (North Coast, Gilroy, CA, USA) (2.0 g, punctate innocuous touch), pinprick without penetrating the skin (noxious pinprick), laser pulse (2 W, 15 ms) generated by an ultra-pulse carbon dioxide laser therapeutic machine (DM-300, Dimei, Changchun, China) (noxious heat). Each type of sensory stimulus was delivered 20 times, with interstimulus intervals of no less than 60 s to avoid hyperalgesia. Percentages of nociceptive behaviors as visualized by flicking, flinching, or licking of the left hind paw were calculated [20, 26].

As the rats developed allodynia after SNI modeling, the nociceptive behaviors became excessive with the accumulation of stimulation times that caused severe movement noise in the signal. In this case, for sham/SNI modeled recording experiments, only von Frey filament (2.0 g) and pinprick stimuli were delivered in the above manner, but confined to the region of the paw innervated by the sural nerve. Electrophysiological data were collected within 1-month post-surgery. The electrode location was confirmed with histology after all recording sessions.

### Data Processing, Spike Sorting, and Unit Classification

The stimulation time points were aligned with the electrophysiological signal and manually calibrated based on the recording video. Trials and channels with noise contamination were discarded.

To measure the spiking activity of single units, raw data were high-pass filtered at 300 Hz. Signals where the amplitude variance exceeded 2 standard deviations were considered to contain multi-unit activity. Spikes were sorted into single units by amplitude and principal components followed by manual adjustment using KlustaKwik (http://klustakwik.sourceforge.net/) [27]. All units were further classified according to previously reported criteria using CellExplorer (https://cellexplorer.org/) [28]. Briefly, units with a wide waveform (trough-to-peak > 0.425 ms) and a bursting firing pattern (the rise time of the auto-correlogram ≤ 6 ms) were identified as putative pyramidal neurons. Units with a spontaneous firing rate < 0.4 Hz were excluded from further analysis.

### Single Unit Activity Analysis

To measure neuronal responses to sensory stimuli, the firing rates of putative pyramidal neurons 2 s before and 2 s after stimulus delivery were calculated and compared using the non-parametric Wilcoxon signed-rank test. The neuronal responses to a sensory stimulus were thus divided into excitatory (increased firing rate with *P* value < 0.05), inhibitory (decreased firing rate with *P* value < 0.05), or neutral (*P* value > 0.05) responses.

To visualize the firing rate variation over time, the spike firing sequences from −5 s to 20 s relative to stimulus delivery were binned in 100-ms epochs. The firing rate of each bin was normalized to the baseline period (5 s before stimulus delivery) for each neuron by z-score: post-stimulus firing rates at each time bin minus baseline average firing rate, divided by the standard variation of baseline firing rate.

To quantify the response magnitudes *via* the z-score changes, the area under the curve (AUC) was calculated by trapezoidal integration, and an absolute value was taken.

To quantify how strongly individual neurons were modulated by sensory stimuli, firing rates were averaged over trials for each type of stimulus, and the modulation index was calculated as follows: | (FR_after_−FR_before_)∕(FR_after_+FR_before_) |. The modulation index ranges from 0 to 1. The closer the value was to 1, the more strongly the neuron was modulated by the sensory stimulus. The value is zero if there is no modulation [20].

### Decoding of Stimuli Based on vCA1 Neuronal Activity

Naïve Bayes classifiers in the MATLAB programming environment (fitcnb) were applied to test the specificity of the vCA1 neural code in response to various stimuli [7]. The goal of a naïve Bayes classifier is to predict the most likely stimulus type based on the firing activity of the recorded vCA1 putative pyramidal neurons.

We constructed a three-dimensional matrix consisting of binned firing rates (100-ms epochs) in a 2-s window after stimulus delivery for each neuron on each trial. The time dimension was then compressed by singular value decomposition. Therefore, the dataset for decoding analysis was a two-dimensional matrix composed of the firing features of all neurons in all trials. We applied 50 rounds of analysis by training a new decoder using a randomly-selected set of training trials (70% of trials) and testing that decoder on a non-overlapping set of test trials (30% of trials) from the entire dataset. The confusion matrix was constructed from the predicted and actual stimuli, each row (corresponding to each actual stimulus) normalized by the number of actual stimuli given to allow comparison of the decoder accuracy. The test sample classification error (*loss*) of the naïve Bayes classifier was estimated to determine how well the algorithm performed. To ensure that the decoding specificity was due to the dynamic neural activity in response to each stimulus, another 50 rounds of decoding analysis were applied using the pre-stimulus firing features. This degraded much of the predicted stimulus specificity. To determine whether different responses of putative pyramidal neurons in vCA1 participate in the encoding of sensory information, the decoding analyses were applied with the firing features of the inhibitory or excitatory response neurons removed from the data set.

### Spectral Analysis

To extract the local field potential (LFP), raw data were down-sampled to 1,250 Hz, low-pass filtered at 200 Hz, and notch-filtered between 48 and 52 Hz. Spectral analysis was applied to averaged LFPs across channels.

For power spectrum analysis, LFP signals during the spontaneous neural activity recording phase were fragmented into 20-s segments and computed using the Chronux toolbox (mtspectrumsegc; http://chronux.org/). The power spectral density was divided into 5 frequency bands: delta (1–4 Hz), theta (4–12 Hz), beta (12–30 Hz), low gamma (30–50 Hz), and high gamma (50–100 Hz).

Theta band power spectrograms from −1 s to 2 s relative to stimulus delivery were constructed by short-time Fourier transform. The power spectrograms were averaged across trials, then normalized to baseline (1 s before stimulus) by dividing the baseline-subtracted power [29].

### Phase-Locking Analysis

To investigate the relationship between single-unit activity and LFP, the distribution of instantaneous phases according to spiking was calculated. To assess nociception-related phase-locking, we analyzed the data 2 s after stimulus onset among all trials of each stimulus for each recorded neuron. First, the LFP signal (recorded from the same tetrode as single units) was 4–12 Hz band-pass filtered using the *FilterLFP* function from the FMAToolbox (https://fmatoolbox.sourceforge.net/). The phase vector of the filtered LFP was then computed using the Hilbert transform. The phase interval from −π to π was uniformly divided into 16 bins and the number of spikes in each phase interval was counted. Neurons with fewer than 40 spikes during the analyzed period were excluded from phase locking estimation. The Rayleigh Z test was applied using the CircStat toolbox to assess the non-uniformity of the spiking phase distribution of each neuron [30]. The neurons were considered significantly phase-locked if *P* < 0.05. The spike-LFP phase vector was computed. The mean resultant length (MRL) was the sum of vector values and ranges from 0 to 1. An MRL value of 1 indicates exact phase synchrony, whereas a value of 0 indicates no phase synchrony.

### Optogenetics

The rat was anesthetized with 1% pentobarbital sodium (50 mg/kg, i.p.) and positioned on a stereotaxic frame (RWD, Shenzhen, China). Adeno-associated virus (AAV) serotype 2/9 carrying the gene for a fusion protein comprised of enhanced halorhodopsin and mCherry fluorescence protein under the calmodulin kinase-II alpha promoter (AAV2/9-CaMKIIα-eNpHR3.0-mCherry; BrainVTA, Wuhan, China) or mCherry alone (AAV2/9-CaMKIIα-mCherry; BrainVTA, Wuhan, China) was bilaterally injected into the vCA1 (AP: −4.9 mm; ML: ±5.5 mm from bregma; DV: −6.0 mm from brain surface) with 0.5 µL/side, at a speed of 0.1 µL/min through a 1-µL microsyringe (RWD, Shenzhen, China). After injection, the needle was left *in situ* for an additional 5 min to allow for solution diffusion. Two optical fibers (OriginOpto, Hangzhou, China) were implanted bilaterally 0.3 mm above the injection sites. Fibers were secured to the skull using 4 stainless steel screws and dental cement.

Behavioral tests were applied 4 weeks after the virus injection. Constant yellow light (589 nm, 6–8 mW) was delivered bilaterally to the vCA1 from an optogenetic system (Newdoon, Hangzhou, China) at the same time as plantar stimulus delivery. The fiber implantation site and virus expression were verified after behavioral testing.

### Chemogenetics

Chemogenetic virus (AAV2/9-CaMKIIα-hM3D(Gq)-mCherry; Vigene Biosciences, Shandong, China) or control vector (AAV2/9-CaMKIIα-mCherry; Vigene Biosciences, Shandong, China) was bilaterally injected into the vCA1 followed the protocol described above. Behavioral tests and electrophysiological recordings were performed 4 weeks after the virus injection.

For chemogenetic activation of vCA1 pyramidal neurons, rats were injected intraperitoneally (1.0 mg/kg dissolved in normal saline) with clozapine-*N*-oxide (CNO; TargetMol) [19]. To verify the efficacy time, electrophysiological recordings were made 1 h before and 1, 8, and 24 h after CNO injection. Average neuronal firing rates were calculated during each 20-min recording session.

Independent cohorts of rats from the electrophysiological recordings were used for behavioral tests. Mechanical pain thresholds were measured 1 day before, and 7, 14, 21, and 28 days after SNI or sham surgery, during the 4–8 h period after CNO injection. Virus expression was verified after behavioral testing.

### Mechanical Pain Thresholds Measurement

Rats were habituated in a transparent plastic box on a metal mesh floor before testing. von Frey filaments (0.41–15.1 g; North Coast, Gilroy, CA, USA) were applied to the lateral plantar surface of the left hind paws (i.e., to the receptive field of the sural nerve). The 50% paw withdrawal threshold was measured by the ‘up and down’ method as previously described [31, 32]. Behavioral tests were carried out single-blindly by another person who was blind in the animal grouping.

### Real-Time Place Preference

The rat in the real-time place preference (RTPP) test was placed in the center of a circular chamber (80 cm in diameter) and allowed to freely explore it for 40 min. The arena was artificially divided into two equal parts along the diameter and one side was paired with constant 589 nm light stimulation. The side receiving light stimulation was counterbalanced [21]. Video recordings from each rat were captured using an overhead camera throughout the test. Rats’ movement tracks were extracted from the videos and the time spent on the stimulation-paired side was measured using SMART software (version 2.5.21, Panlab, SMART Video tracking, Harvard Apparatus). The chamber was cleaned with 75% ethanol between tests.

### Anatomical Orientation and Histology

The rat was deeply anesthetized with 1% pentobarbital sodium (50 mg/kg, i.p.) and perfused intracardially with 0.9% saline followed by 4% paraformaldehyde (PFA, in 0.1 M phosphate buffer, pH 7.4). The isolated brain was post-fixed in 4% PFA for at least 12 h, and dehydrated in 20% and 30% sucrose solutions in turn. The fixed brain was cut coronally at 40 µm on a cryostat microtome (CM1950, Leica, Germany).

Recording sites were identified by visual examination of electrolytic lesions, which were induced immediately before perfusion by passing current (2 mA, 15 s) through the electrode [20]. The brain slices were stained with Nissl solution and photographed under a light microscope (Leica DMI 4000B, Wetzlar, Germany).

Virus infection was validated after behavioral tests. Brain slices were mounted after incubation with a DNA-specific fluorescent probe (DAPI: 4′,6-diamidino-2-phenylindole, 1:1,000, Cell Signaling Technology). Fluorescence images were acquired using a laser scanning confocal microscope (model FV1000, Olympus Co., Ltd.)

### Statistical Analysis

Statistical analyses were performed using GraphPad Prism 8 or MATLAB. Wilcoxon signed-rank tests or Mann-Whitney tests were used to compare neuronal firing rates. Chi-squared tests were used to compare proportions. Group comparisons were made using either one-way or two-way analysis of variance (ANOVA) followed by the Bonferroni *post hoc* test for parametric statistics, and the Kruskal-Wallis test with Dunn’s *post hoc* test for nonparametric statistics. Single variable comparisons were made with two-tailed paired or unpaired Student’s *t*-tests. Pearson correlation tests were used to analyze the correlation between variables. Data are expressed as the mean ± SEM, with *P* < 0.05 as statistically significant.

## Results

### vCA1 Pyramidal Neuronal Activity is Strongly Modulated by Nociceptive Stimulation

**Fig. 1:**
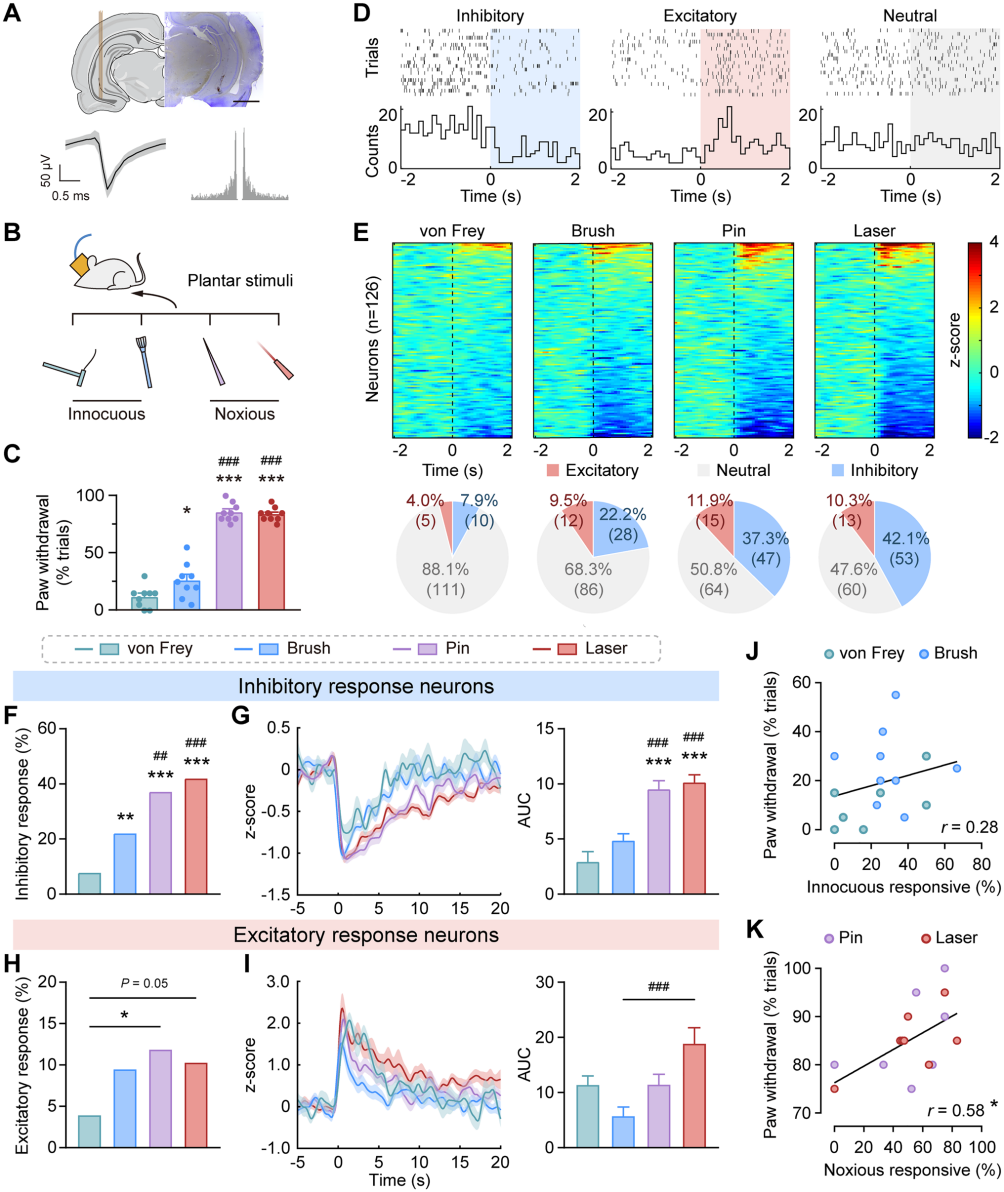
vCA1 pyramidal neuronal activity is strongly modulated by nociceptive stimulation. **A** Upper, representative image of a recording track in the vCA1 (scale bar, 2 mm). Lower, representative waveform and auto-correlogram of a putative pyramidal neuron. **B** Schematic of the experimental paradigm. **C** Noxious stimuli induce stronger paw withdrawal responses than innocuous stimuli. 9 recording sessions from 3 rats. *P < 0.05, ***P < 0.001 *vs* von Frey; ^###^*P* < 0.001 *vs* brush, one-way ANOVA with Bonferroni’s *post hoc* test. **D** Representative raster plots and peri-stimulus time histograms (PSTHs) of the inhibitory, excitatory, and neutral response of vCA1 putative pyramidal neurons to plantar stimuli. Examples from laser stimulation. **E** Stimulus-evoked responses of vCA1 putative pyramidal neurons. Heatmaps represent the z-score normalized PSTHs for individual neurons relative to stimuli onset (100-ms bins). Pie charts show the proportions of neurons responding to each stimulus. **F** Statistical analyses of the proportion of inhibitory responses. **P < 0.01, ***P < 0.001 *vs* von Frey; ^##^*P* < 0.01, ^###^*P* < 0.001 *vs* brush, Chi-squared test. **G** Left, the averaged z-score of inhibitory response neurons. Right, response magnitude quantified by AUC (area under the curve). ***P < 0.001 *vs* von Frey; ^###^*P* < 0.001 *vs* brush, one-way ANOVA with Bonferroni’s *post hoc* test. **H** Statistical analyses of the excitatory response proportions. *P < 0.05 *vs* von Frey, Chi-squared test. **I** The averaged z-score (left) and response magnitude (AUC, right) of excitatory response neurons to plantar stimuli. ^###^*P* < 0.001 *vs* brush, one-way ANOVA with Bonferroni’s *post hoc* test. **J** No correlation between the neuronal response ratio and withdrawal ratio to innocuous stimuli. **K** Positive correlation between the neuronal response ratio and withdrawal ratio to noxious stimuli. *P < 0.05, Pearson correlation test.

To characterize the recruitment and firing modulation of vCA1 neurons during peripheral sensory stimulation, we made electrophysiological recordings in the vCA1 of freely-behaving rats presented with diverse plantar stimuli, including a 2.0-g von Frey filament (punctate), brush (dynamic), pinprick (mechanical), and laser (thermal) (Fig. 1A, B). Pinprick and laser stimulation induced significantly stronger withdrawal behaviors, and were therefore referred to as noxious stimuli. Although the individual responses to dynamic tactile stimuli varied, von Frey and brush did not induce notable withdrawal behaviors, and were further referred to as innocuous stimuli (*F*_(3,32)_ = 127.20, *P* < 0.001, one-way ANOVA with Bonferroni’s *post hoc* test; Fig. 1C).

A total of 182 single units in the vCA1 were recorded, among which 126 were putative pyramidal neurons based on their waveforms and firing properties (Fig. 1A). Putative pyramidal neurons that showed a significant firing rate decrease or increase in response to plantar stimuli were classified as inhibitory or excitatory response neurons, respectively (Wilcoxon signed-rank test, *P* < 0.05; Fig. 1D). Neurons with no significant firing rate change were classified as neutral (Wilcoxon signed-rank test, *P* ≥ 0.05; Fig. 1D).

We found a significantly greater proportion of vCA1 putative pyramidal neurons showed an inhibitory response to noxious stimuli than innocuous stimuli (brush *vs* von Frey, *χ*^2^_(1)_ = 10.04, *P* < 0.01; pin *vs* von Frey, *χ*^2^_(1)_ = 31.04, *P* < 0.001; laser *vs* von Frey, *χ*^2^_(1)_ = 39.13, *P* < 0.001; pin *vs* brush, *χ*^2^_(1)_ = 6.85, *P* < 0.01; laser *vs* brush, *χ*^2^_(1)_ = 11.37, *P* < 0.001; laser *vs* pin, *χ*^2^_(1)_ = 0.60, *P* > 0.05, Chi-squared test; Fig. 1E, F), along with a potentiated change in response magnitude (*F*_(3,134)_ = 14.46, *P* < 0.001, one-way ANOVA with Bonferroni’s *post hoc* test; Fig. 1G). Similarly, albeit smaller, the proportion of neurons that exhibited an excitatory response to noxious stimuli was higher than to a von Frey stimulus (brush *vs* von Frey, *χ*^2^_(1)_ = 3.09, *P* > 0.05; pin *vs* von Frey, *χ*^2^_(1)_ = 5.43, *P* < 0.05; laser *vs* von Frey, *χ*^2^_(1)_ = 3.83, *P* = 0.05; pin *vs* brush, *χ*^2^_(1)_ = 0.37, *P* > 0.05; laser *vs* brush, *χ*^2^_(1)_ = 0.04, *P* > 0.05; laser *vs* pin, *χ*^2^_(1)_ = 0.16, *P* > 0.05, Chi-squared test; Fig. 1E, H). However, as the number of the excitatory response neurons was too small, we failed to find any regular changes in the magnitude of their response to stimuli (*F*_(3,41)_ = 6.48, *P* < 0.01, one-way ANOVA, Bonferroni’s *post hoc* multiple comparisons indicated only a significant difference between brush *vs* laser: *P* < 0.001 in all cases; Fig. 1l).

To verify that the change in firing rate of vCA1 putative pyramidal neurons was caused by nociception rather than retraction, we analyzed the correlation between withdrawal ratio and neuronal response ratio for innocuous and noxious stimuli (Fig. 1J, K). Notably, the proportion of noxious stimuli-responsive neurons positively correlated with the paw withdrawal ratio (*r* = 0.58, *P* < 0.05, Pearson correlation test; Fig. 1K). However, the proportion of innocuous stimuli-responsive neurons did not correlate with withdrawal behaviors (*r* = 0.28, *P* > 0.05, Pearson correlation test; Fig. 1J). These results indicate that responses of vCA1 putative pyramidal neurons to sensory stimuli are induced by nociception, but not non-specific withdrawal movements.

**Fig. 2:**
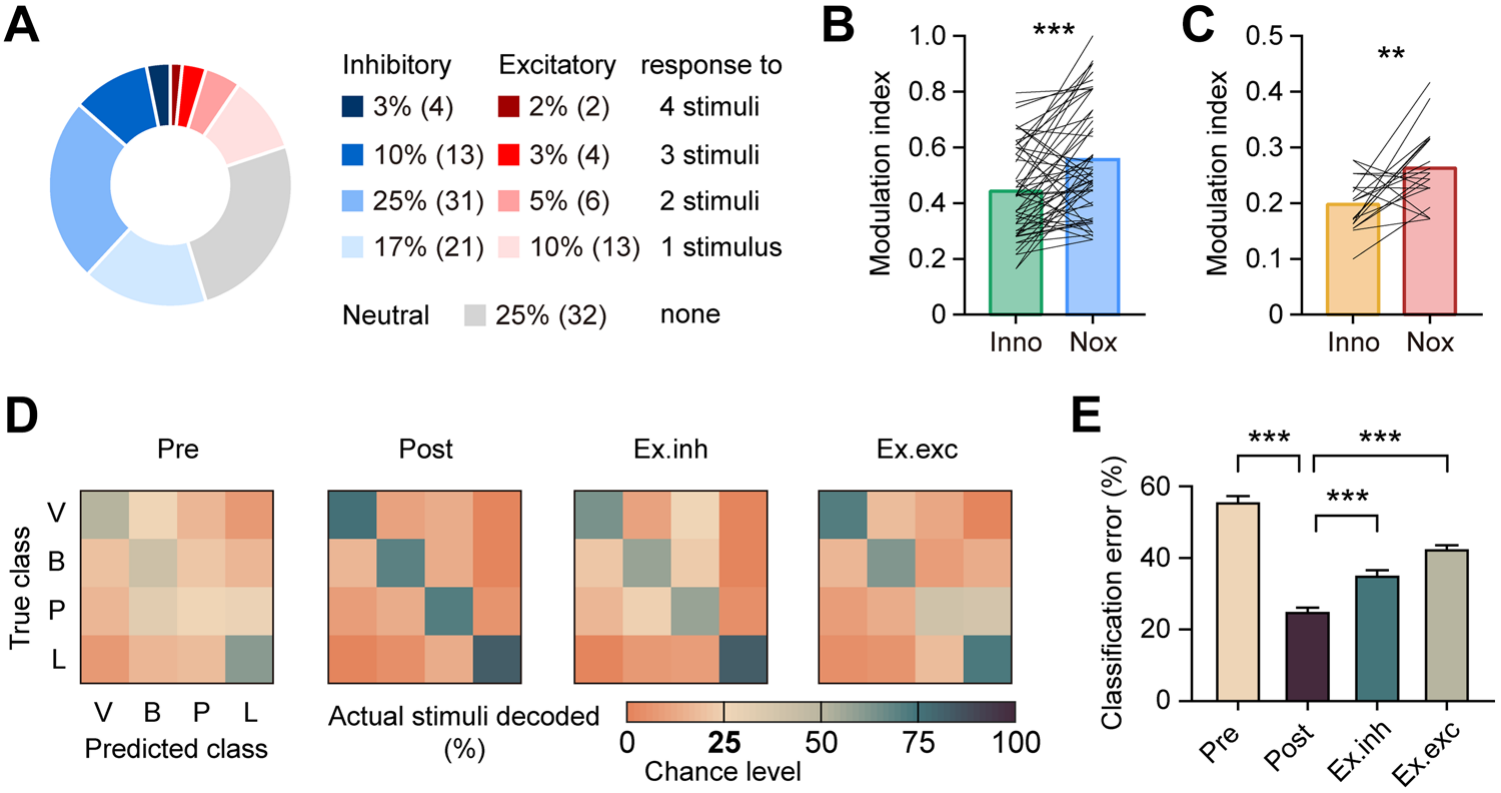
vCA1 pyramidal neuronal activity encodes stimulus modalities. **A** Among the 126 putative pyramidal neurons recorded in the vCA1, 3%, 10%, 25%, and 17% show an inhibitory response to 4–1 types of stimulus (blue, from dark to light); 2%, 3%, 5%, and 10% show an excitatory response to 4–1 types of stimulus (red, from dark to light), and 25% show a neutral response to all of the 4 stimuli (gray). **B, C** Multi-stimuli inhibitory (**B**) and excitatory (**C**) responsive pyramidal neurons are modulated more strongly by noxious stimuli (Nox) than innocuous stimuli (Inno). **P < 0.01, ***P < 0.001, paired *t*-test. **D** Confusion matrixes of the predicted and actual stimuli. From left to right: decoders trained and tested by data from 2 s before stimuli (Pre), data from 2 s after stimuli (Post), post-stimulation data excluding inhibitory response features (Ex.inh), post-stimulation data excluding excitatory response features (Ex.exc). Class abbreviations: V, von Frey; B, brush; P, pin; L, laser. **E** The decoding performance as measured by classification error. ***P < 0.001, Kruskal-Wallis test with Dunn’s *post hoc* test.

Next, we analyzed how individual vCA1 putative pyramidal neurons responded to various sensory stimuli. 55% of the recorded neurons showed an inhibitory response and 20% showed an excitatory response to one or more types of stimulus, with 25% responding to none of the 4 kinds of stimulus (Fig. 2A). Surprisingly, none of the recorded neurons showed a change in firing rate in opposite directions in response to different stimuli, indicating that the neurons with inhibitory and excitatory responses were separate subsets of vCA1 neurons. The neurons responsive to multiple stimuli were modulated more strongly by noxious stimuli than innocuous ones, regardless of the response category (inhibitory response: *t*_(54)_ = 4.52, *P* <0.001, paired t-test; Fig. 2B, and excitatory response: *t*_(18)_ = 3.21, *P* < 0.01, paired t-test; Fig. 2C).

Due to the diversity of response category, proportion, and intensity of vCA1 putative pyramidal neurons to different stimuli, we conjectured that the vCA1 activity pattern encodes sensory information. To test this hypothesis, we applied a naïve Bayes classifier to decode stimulus types from matrices of firing features of the recorded vCA1 putative pyramidal neurons. The classifier trained by data from 2 s after stimulation was able to distinguish each stimulus with high accuracy, indicating that sensory stimuli of different intensities and modalities were represented by unique activity codes in the vCA1 (Fig. 2D, Post). As the neuronal activity before stimulation did not possess sensory information, the classifier trained by data from 2 s before stimuli as control degraded much of the prediction specificity (Fig. 2D, Pre). To determine whether the inhibitory and excitatory responses of vCA1 putative pyramidal neurons contribute to sensory information encoding, we removed the inhibitory or excitatory response features from the post-stimulation data and ran the decoding analysis again (Fig. 2D, Ex.inh and Ex.exc). Surprisingly, removing either type of response feature led to worse decoding performance compared with decoding by the full dataset, but still much better than decoding by the pre-stimulation dataset (Kruskal-Wallis statistic = 118.50, *P* <0.001, Kruskal-Wallis test with Dunn’s *post hoc* test; Fig. 2E). Taken together, these findings indicate that both decreased and increased activity of putative pyramidal neurons after stimulation are necessary for sensory encoding in vCA1.

### vCA1 Pyramidal Neurons with Inhibitory or Excitatory Responses to Stimuli Differentially Contribute to Nociceptive Information Encoding During Neuropathic Pain

**Fig. 3:**
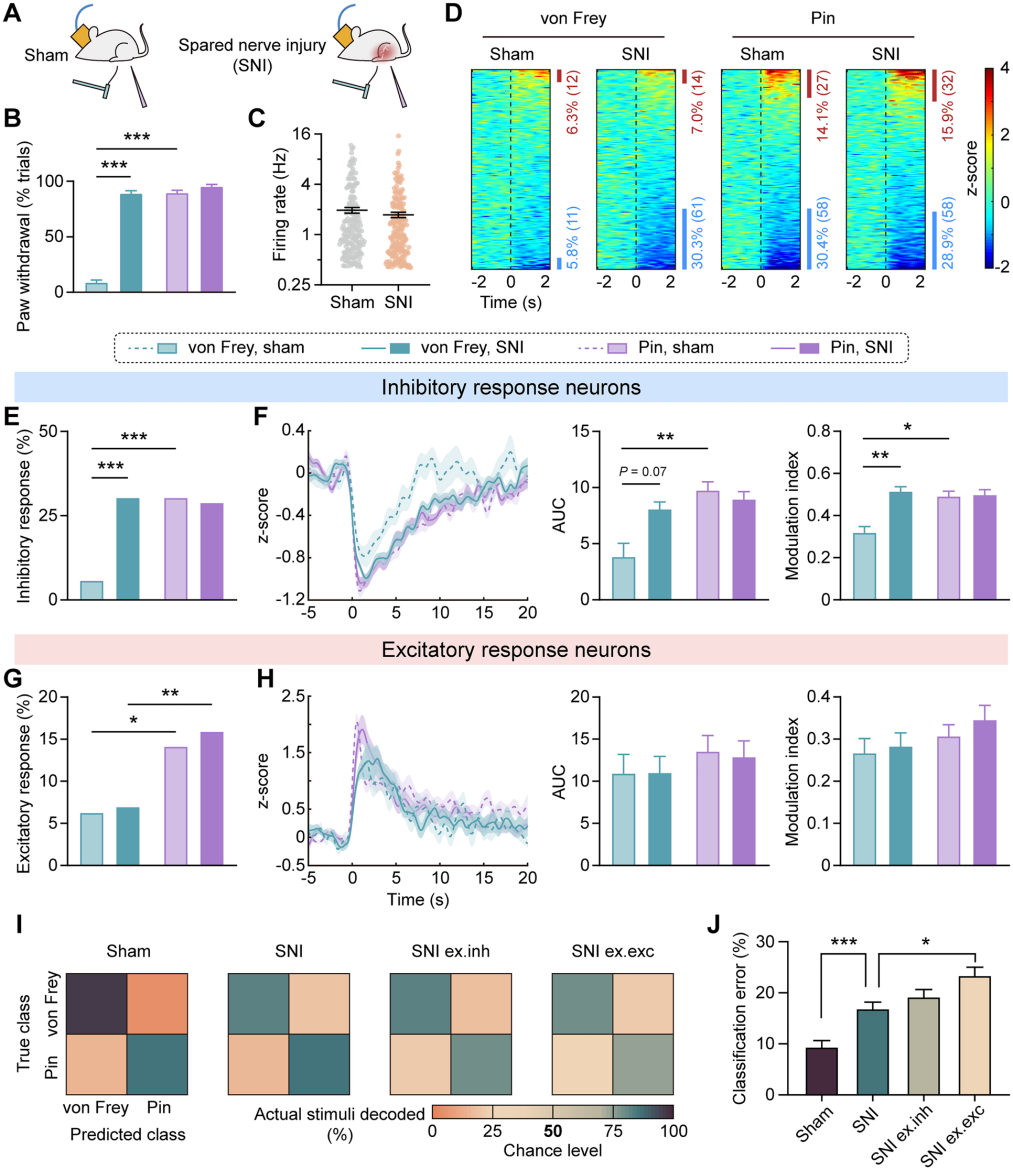
vCA1 pyramidal neurons with inhibitory or excitatory responses to stimuli differentially contribute to nociceptive information encoding during neuropathic pain. **A** Schematic of the experimental paradigm. **B** Behavioral responses to plantar stimuli in the sham (19 recording sessions from 5 rats) and SNI (22 recording sessions from 4 rats) groups. ***P <0.001, two-way ANOVA with Bonferroni’s *post hoc* test. **C** Spontaneous firing rates of vCA1 putative pyramidal neurons in sham (*n* = 191 neurons) and SNI (*n* = 201 neurons) groups. **D** Stimulus-evoked responses of vCA1 putative pyramidal neurons. Heatmaps represent the z-score normalized PSTHs for individual neurons relative to stimulus onset (100-ms bins). Bars indicate the percentages of neurons with inhibitory (blue) or excitatory (red) responses to stimuli. **E** Statistical analyses of the inhibitory response proportion. ***P < 0.001, Chi-squared test. **F** The averaged z-score of inhibitory response neurons (left). Response magnitude quantified by AUC (middle) and modulation index (right). *P < 0.05, **P < 0.01, two-way ANOVA with Bonferroni’s *post hoc* test. **G** Statistical analyses of the excitatory response proportion. *P < 0.05, **P < 0.01, Chi-squared test. **H** The averaged z-score (left) and response magnitude (AUC, middle; modulation index, right) of excitatory response neurons to plantar stimuli. **I** Confusion matrixes of the predicted and actual stimuli. From left to right: decoder trained by the firing features 2 s after stimulation from the sham group (Sham), SNI group (SNI), SNI group excluding inhibitory response features (SNI ex.inh), and SNI group excluding excitatory response features (SNI ex.exc). **J** The decoding performance as measured by classification error. *P < 0.05, ***P < 0.001, Kruskal-Wallis test with Dunn’s *post hoc* test.

We further investigated how vCA1 putative pyramidal neurons of different response categories participate in nociceptive information encoding in the SNI model of neuropathic pain rats. 2.0-g von Frey filament and pinprick stimulation were delivered to the lateral surface of the left hind paw of sham or SNI rats during electrophysiological recording (Fig. 3A). A hallmark of chronic neuropathic pain is the appearance of allodynia, in which innocuous stimuli evoke nociceptive responses just like noxious stimuli [2]. Indeed, the nerve-injured rats displayed significantly more severe nociceptive responses to the 2.0-g von Frey filament compared with sham group, nearly the same level of noxious pinprick (group effect: *F*_(1,78)_ = 432.50, *P* < 0.001; stimulus effect: *F*_(1,78)_ = 441.00, *P* < 0.001; interaction: *F*_(1,78)_ = 325.30, *P* < 0.001, two-way ANOVA with Bonferroni’s *post hoc* test; Fig. 3B).

The spontaneous activity of vCA1 putative pyramidal neurons did not differ between sham and SNI groups (*U* = 18016, *P* >0.05, Mann-Whitney test. Fig. 3C). In the sham group, we found a larger proportion of vCA1 putative pyramidal neurons were inhibited (von Frey–sham *vs* pin–sham, *χ*^2^_(1)_ = 39.07, *P* < 0.001, Chi-squared test; Fig. 3E) and more strongly modulated by pin prick than von Frey stimuli (AUC: group effect: *F*_(1,184)_ = 3.18, *P* > 0.05; stimulus effect: *F*_(1,184)_ = 12.33, *P* < 0.001; interaction: *F*_(1,184)_ = 6.81,* p* < 0.01; *P* < 0.01 for von Frey–sham *vs* pin–sham; modulation index: group effect: *F*_(1,184)_ = 9.83, *P* < 0.01; stimulus effect: *F*_(1,184)_ = 5.83, *P* < 0.05; interaction: *F*_(1,184)_ = 8.42, *P* < 0.01; *P* < 0.05 for von Frey–sham *vs* pin–sham, two-way ANOVA with Bonferroni’s *post hoc* test; Fig. 3F), which paralleled the above findings. However, the proportion of pyramidal neurons with an inhibitory response to von Frey stimulation was significantly greater for the SNI group than the sham group (von Frey–sham *vs* von Frey–SNI, *χ*^2^_(1)_ = 39.49, *P* < 0.001, Chi-squared test; Fig. 3E), as well as the modulation magnitude (AUC: *P* = 0.07; modulation index: *P* < 0.01 for von Frey–sham *vs* von Frey–SNI; Fig. 3F). There was no significant difference between the proportion (von Frey–SNI *vs* pin–SNI, *χ*^2^_(1)_ = 0.11, *P* > 0.05, Chi-squared test; Fig. 3E) and modulation magnitude (AUC and modulation index: *P* > 0.05 for von Frey–SNI *vs* pin–SNI; Fig. 3F) of neurons with an inhibitory response to von Frey or pin in the SNI group. In contrast, more neurons elicited an excitatory response to pinprick than von Frey stimulation in both normal and neuropathic pain states (von Frey–sham *vs* pin–sham, *χ*^2^_(1)_ = 6.43, *P* < 0.05; von Frey–SNI *vs* pin–SNI, *χ*^2^_(1)_ = 7.95, *P* < 0.01, Chi-squared test; Fig. 3G), though without significant changes in modulation magnitude (AUC: group effect: *F*_(1,81)_ = 0.02, *P* > 0.05; stimulus effect: *F*_(1,81)_ = 0.99, *P* > 0.05; interaction: *F*_(1,81)_ = 0.03, *P* > 0.05; modulation index: group effect: *F*_(1,81)_ = 0.53, *P* > 0.05; stimulus effect: *F*_(1,81)_ = 1.87, *P* > 0.05; interaction: *F*_(1,81)_ = 0.09, *P* > 0.05, two-way ANOVA with Bonferroni’s *post hoc* test; Fig. 3H). These results indicate an intriguing finding that, among the vCA1 putative pyramidal neurons, the response proportion and magnitude of those inhibited by plantar stimuli closely parallel the nociceptive response, whereas the proportion of those excited by stimuli mirrors the actual intensity of those stimuli.

To further confirm the conclusion above, we applied the decoding analysis using firing features of vCA1 putative pyramidal neurons 2 s after stimulation from sham and SNI groups (Fig. 3I). The classification errors of the classifiers trained by SNI datasets were markedly higher than classifiers trained by sham datasets, indicating confounded encoding of innocuous and noxious stimuli during neuropathic pain. Removing the inhibitory response features from SNI datasets did not worsen the decoding performance, suggesting that these features no longer contributed to the classification of innocuous and noxious stimuli during neuropathic pain. However, neurons excited after stimulation still played a prominent role in the encoding, as the classification error increased when removing the excitatory response features from SNI datasets (Kruskal-Wallis statistic = 39.87, *P* < 0.001, Kruskal-Wallis test with Dunn’s *post hoc* test; Fig. 3J). Taken together, the decoding results further demonstrate the functional heterogeneity of the stimulus-evoked vCA1 neuronal activity in neuropathic pain, in which the inhibitory response neurons fail to discriminate between innocuous and noxious stimuli and may therefore contribute to allodynia, yet the excitatory response neurons help to accomplish the differentiation of stimuli properties.

### Ventral Hippocampal Theta Oscillation in Response to Nociception

**Fig. 4:**
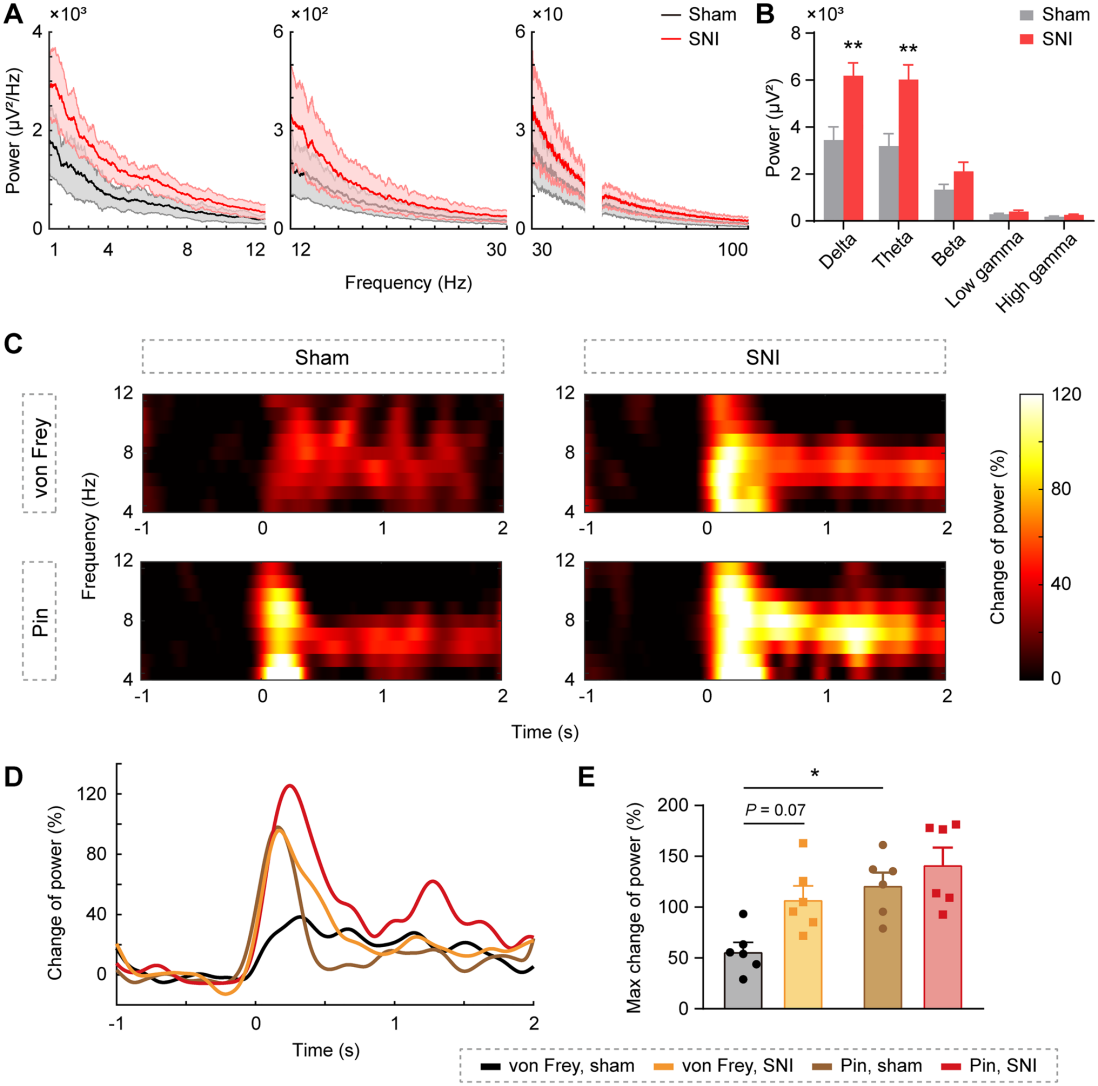
The ventral hippocampal theta oscillation in response to nociception. **A, B** Enhanced LFP power in the delta and theta bands of vCA1 during neuropathic pain. Frequency bands: delta (1–4 Hz), theta (4–12 Hz), beta (12–30 Hz), low gamma (30–50 Hz), and high gamma (50–100 Hz). *n* = 6 per group. **P < 0.01, unpaired *t*-test. **C** Theta band power spectrograms of vCA1 in response to von Frey and pin stimulation (rows) in sham and SNI groups (columns). **D, E** Increased increment of theta power to pin stimulation in the sham group and both stimuli in the SNI group. *P < 0.01, two-way ANOVA with Bonferroni’s *post hoc* test.

The LFP reflects the coordination of neural activity in local brain regions [33]. Theta is a prominent oscillation type of the hippocampus, and it has been proposed that theta activation in the dorsal hippocampal CA1 (dCA1) parallels formalin nociception [34]. We wondered if the theta activity in the vCA1 is also involved in nociception. Power spectral density (PSD) analysis of the vCA1 in the resting state revealed enhanced power in the delta and theta bands during neuropathic pain (delta: *t*_(10)_ = 3.48, *P* < 0.01; theta: *t*_(10)_ = 3.43, *P* < 0.01; beta: *t*_(10)_ = 1.73, *P* > 0.05; low gamma: *t*_(10)_ = 1.60, *P* > 0.05; high gamma: *t*_(10)_ = 1.62, *P* > 0.05; unpaired *t*-test; Fig. 4A, B). Pinprick stimulation induced considerably higher theta enhancement than von Frey in the sham group, while both stimuli evoked robust theta activation in the SNI group (group effect: *F*_(1,20)_ = 7.47, *P* < 0.05; stimulus effect: *F*_(1,20)_ = 14.51, *P* < 0.01; interaction: *F*_(1,20)_ = 1.40, *P* > 0.05, two-way ANOVA with Bonferroni’s *post hoc* test; Fig. 4C–E). These results demonstrate that an increased increment of theta power in the vCA1 is associated with nociception.

### Nociception Is Characterized by the Synchronization of vCA1 Pyramidal Neuronal Activity and Theta Oscillation

**Fig. 5:**
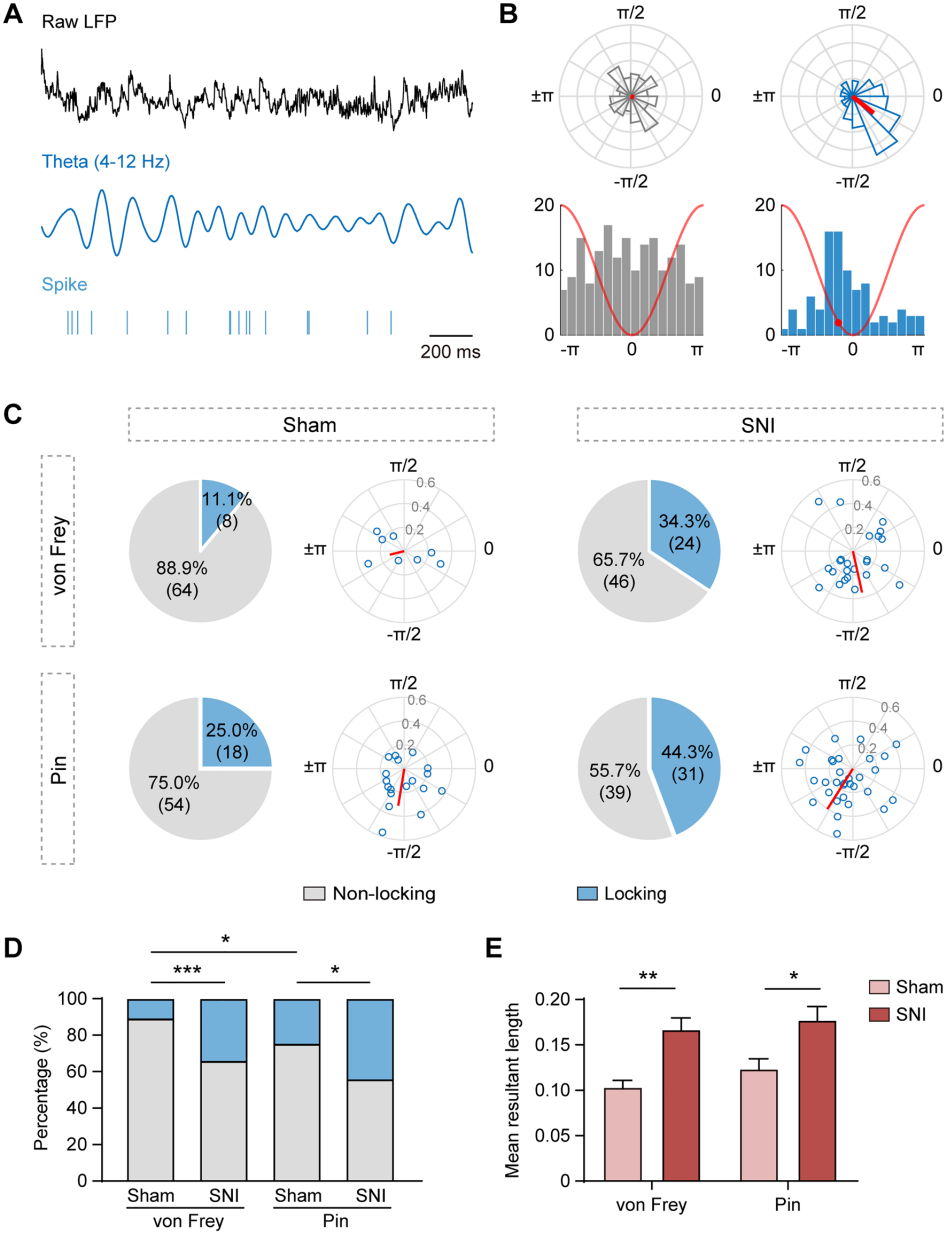
Nociception is characterized by synchronization of vCA1 pyramidal neuronal activity and theta oscillation. **A** Raw vCA1 LFP, theta (4–12 Hz) filtered vCA1 LFP, and representative raster plot of a putative pyramidal neuron spiking in phase with simultaneously recorded vCA1 theta oscillation. **B** Examples of a non-phase locking neuron (left) and a phase locking neuron (right) in the vCA1 relative to local theta oscillation. Polar histograms illustrate the Rayleigh test standard (upper), and vectors in red represent the direction (angle) and magnitude (length) of the mean resultant length (MRL). Histograms show the distribution of spike-phase angles (16 bins per cycle, lower). Red line, one schematic theta cycle; red dot, preference phase angle. **C** Ratios of putative pyramidal neurons locked to vCA1 theta phase during stimulus-evoked responses in sham (*n* = 72 neurons) and SNI (*n* = 70 neurons) groups (pie charts). Preferred theta phase (polar angle) and locking strength (polar radius) for phase-locking neurons (polar scatter plots). **D** An increased proportion of stimulus-evoked phase-locking in vCA1 is related to nociception. *P < 0.05, ***P < 0.001, Chi-squared test. **E** Increased strength in phase-locking of vCA1 neurons to local theta oscillations during neuropathic pain. *P < 0.05, **P < 0.01, two-way ANOVA with Bonferroni’s *post hoc* test.

To determine whether the firing of vCA1 putative pyramidal neurons synchronizes the potentiated theta activity coupled to nociception, we investigated the proportion and strength of phase locking of the vCA1 neurons to local theta oscillation during the 2 s after stimulus delivery (Fig. 5A, B). Upon nociceptive stimulation (i.e. pinprick in the sham group, and both von Frey and pinprick in the SNI group), a larger proportion of vCA1 putative pyramidal neurons fired preferentially in specific phases of local theta oscillation, noting that for the same type of stimulus, the proportion of phase locking in the SNI group was greater relative to the sham group (von Frey–sham *vs* pin–sham, *χ*^2^_(1)_ = 4.69, *P* < 0.05; von Frey–sham *vs* von Frey–SNI, *χ*^2^_(1)_ = 10.92, *P* < 0.001; pin–sham *vs* pin–SNI, *χ*^2^_(1)_ = 5.84, *P* < 0.05; von Frey–SNI *vs* pin–SNI, *χ*^2^_(1)_ = 1.47, *P* > 0.05, Chi-squared test; Fig. 5C, D). Meanwhile, increased phase locking strength measured by the MRL was also found in the SNI group for both stimuli compared with that in the sham group (group effect: *F*_(1,280)_ = 23.76, *P* < 0.001; stimulus effect: *F*_(1,280)_ = 1.62, *P* > 0.05; interaction: *F*_(1,280)_ = 0.17, *P* > 0.05, two-way ANOVA with Bonferroni’s *post hoc* test; Fig. 5E). These findings indicate enhanced theta modulation of evoked vCA1 neuronal activity is associated with nociception.

### Optogenetic Inhibition of vCA1 Pyramidal Neurons Induces Mechanical Allodynia in Naïve Rats

**Fig. 6:**
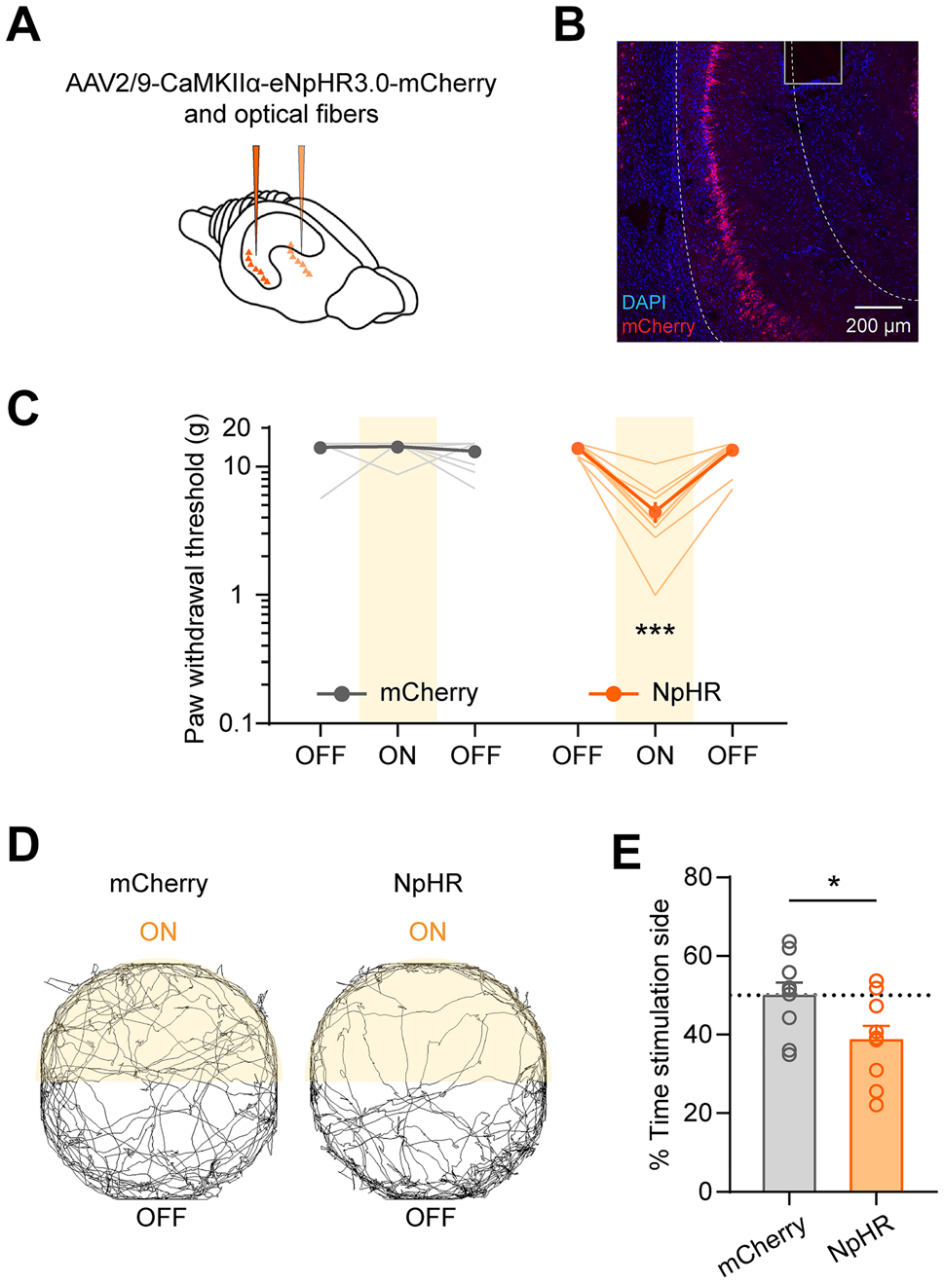
Optogenetic inhibition of vCA1 pyramidal neurons induces mechanical allodynia in naïve rats. **A** Schematic of optogenetic virus injection and optical fiber implantation in the vCA1.** B** Representative confocal image of virus expression and optical fiber location in the vCA1. **C** Inhibition of vCA1 pyramidal neurons induces mechanical allodynia. *n* = 10 per group. ***P < 0.001, two-way ANOVA with Bonferroni’s *post hoc* test.** D** Representative movement tracks in a 10-min period during RTPP from mCherry (left) and NpHR (right) groups. The yellow areas indicate the light stimulation side. **E** Optogenetic inhibition decreases the time spent on the stimulation side in the NpHR group. *n* = 10 per group. *P < 0.05, unpaired *t*-test.

Our electrophysiology results suggest that the inhibitory response of vCA1 putative pyramidal neurons is relevant to nociception and might be the cause of mechanical allodynia. To further verify the causality of this conclusion, we expressed halorhodopsin (NpHR) in bilateral vCA1 pyramidal neurons using adeno-associated viral vector serotype 2/9 (AAV2/9) under the control of the glutamatergic calmodulin kinase-II alpha (CaMKIIα) promoter (Fig. 6A, B). Constant yellow light with a wavelength of 589 nm and von Frey stimulation were delivered simultaneously to mimic the inhibitory response of vCA1 pyramidal neurons. Indeed, inhibition of the vCA1 pyramidal neurons induced mechanical allodynia in naïve rats (group effect: *F*_(1,18)_ = 12.93, *P* < 0.01; light effect: *F*_(2,36)_ = 26.34, *P* < 0.001; interaction: *F*_(2,36)_ = 34.66, *P* < 0.001, two-way ANOVA with Bonferroni’s *post hoc* test; Fig. 6C). Moreover, the NpHR group spent less time on the side with light stimulation in the RTPP assay, indicating increased aversion during vCA1 pyramidal neuron inhibition (*t*_(18)_ = 2.51, *P* < 0.05; unpaired *t*-test; Fig. 6D–E). Together, these results validate the causal role of the inhibitory response of vCA1 pyramidal neurons in mechanical allodynia.

### Chemogenetic Activation of vCA1 Pyramidal Neurons Alleviates Mechanical Allodynia in Chronic Neuropathic Pain

**Fig. 7:**
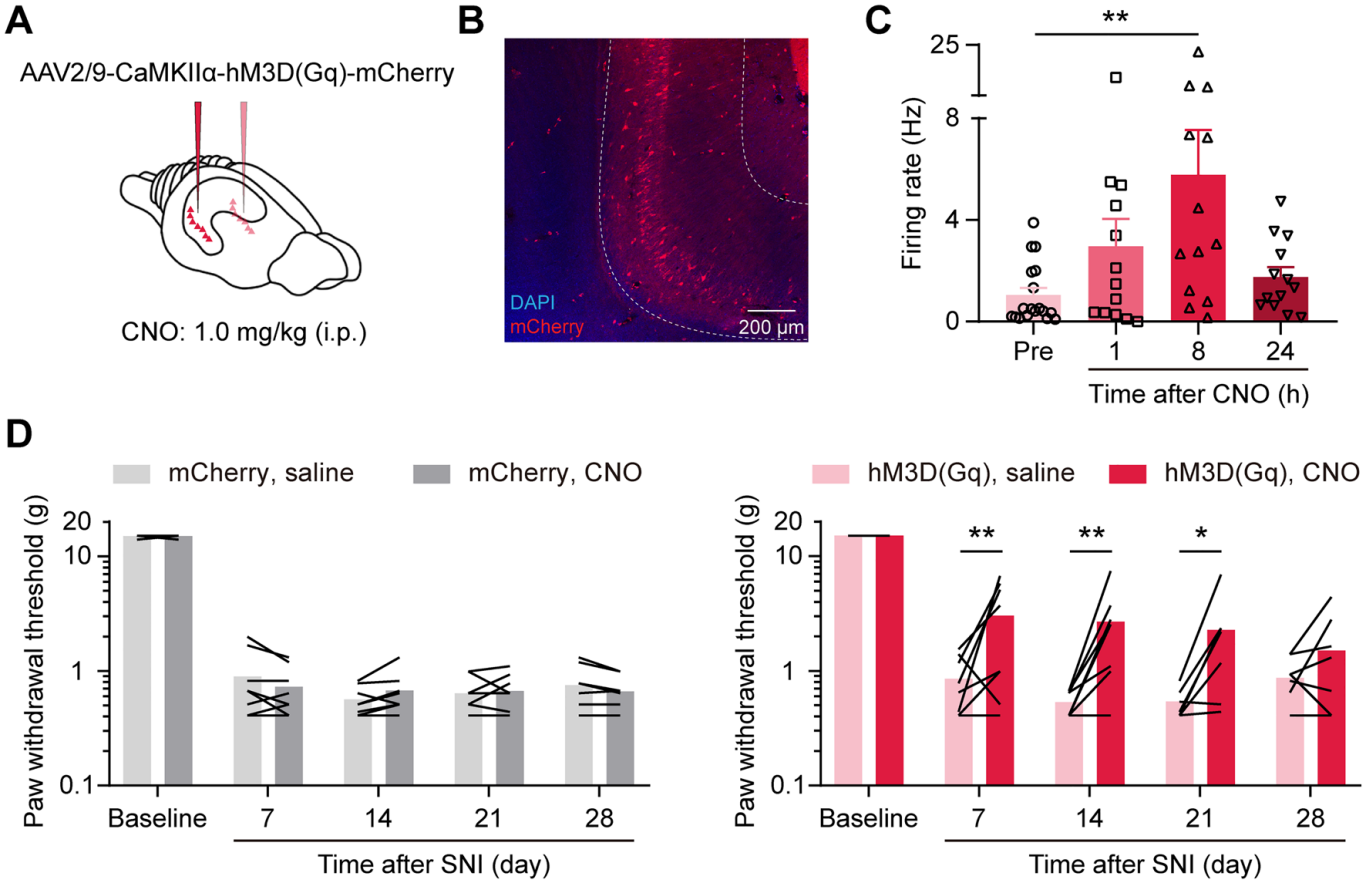
Chemogenetic activation of vCA1 pyramidal neurons alleviates mechanical allodynia in chronic neuropathic pain. **A** Schematic of chemogenetic virus injection in the vCA1; CNO is delivered intraperitoneally. **B** Representative confocal image of virus expression in the vCA1. **C** Increased firing rate of vCA1 putative pyramidal neurons after CNO injection. **P < 0.01, Kruskal-Wallis test with Dunn’s *post hoc* test. **D** Chemogenetic activation of vCA1 pyramidal neurons attenuates SNI-induced mechanical allodynia. *n* = 8 per group. *P < 0.05, **P < 0.01, two-way ANOVA with Bonferroni’s *post hoc* test.

To determine whether the overall inhibited activity of vCA1 pyramidal neurons mediates mechanical allodynia in neuropathic pain, we transfected bilateral vCA1 pyramidal neurons with a virus containing hM3D(Gq), an artificially designed receptor selectively activated by the designed drug CNO (Fig. 7A, B). CNO injection effectively increased the firing rates of vCA1 pyramidal neurons (Kruskal-Wallis statistic = 10.42, *P* < 0.05, Kruskal-Wallis test with Dunn’s *post hoc* test; Fig. 7C). Chemogenetic activation of vCA1 pyramidal neurons attenuated the SNI-induced mechanical allodynia (vector: group effect: *F*_(1,7)_ = 0.23, *P* > 0.05; time effect: *F*_(4,28)_ = 5096.00, *P* < 0.001; interaction: *F*_(4,28)_ = 0.81, *P* > 0.05; hM3D(Gq): group effect: *F*_(1,7)_ = 15.81, *P* < 0.01; time effect: *F*_(4,28)_ = 336.30, *P* < 0.001; interaction: *F*_(4,28)_ = 2.53, *P* > 0.05, two-way ANOVA with Bonferroni’s *post hoc* test; Fig. 7D). These results suggest that the inhibited vCA1 activity is responsible for the abnormal nociception during neuropathic pain.

## Discussion

### Representation of Nociceptive Information in vCA1

In the present study, we discovered that a considerable fraction of vCA1 putative pyramidal neurons exhibits an inhibitory response to noxious stimuli, while a relatively small fraction shows an excitatory response (Fig. 1). These findings are consistent with the previous conclusion that the overall neuronal activity of vCA1 is suppressed in pain [11, 19]. It is worth noting that the noxious-evoked vCA1 responses are unlikely to be attributable to anxiety or fear, as both conditions elicit mainly excitatory responses of the vCA1 pyramidal neurons [21, 22]. The unaugmented proportion of the excitatory response neurons to innocuous stimuli in neuropathic pain-induced allodynia is further evidence for the statement above (Fig. 3G).

Nociceptive information is detected by peripheral nociceptors and subsequently distributed to the spinal dorsal horn and then to numerous brain regions [1]. A growing number of research has shown that the ability to distinguish between sensory modalities is not a unique trait of the sensory cortices [7, 35]. A previous study has demonstrated an amygdalar neuronal ensemble that encodes a variety of thermal and mechanical nociceptive stimuli [7]. Here, we provide evidence that somatosensory stimuli of different intensities and modalities can be distinguished from one another by specific firing features of vCA1 putative pyramidal neurons (Fig. 2). This means that the pyramidal neurons of vCA1, as a whole, are able to encode peripheral sensory information. These findings enrich the representations of sensory information in the limbic system and further emphasize the prominent role of the ventral hippocampus in pain perception.

## Functional Heterogeneity of Stimulus-evoked Inhibitory and Excitatory Responses of Pyramidal Neurons in vCA1

Based on the behavioral characteristics of mechanical allodynia in neuropathic pain, we discovered a functional separation of the inhibited and excited vCA1 pyramidal neurons after stimuli. The proportion and magnitude of inhibitory response neurons paralleled the nociceptive behavior, and these features are implicated in the confounded encoding of innocuous and noxious stimuli during neuropathic pain (Fig. 3). Mechanical allodynia induced by optogenetic inhibition of vCA1 pyramidal neurons in naïve rats further confirmed the causal role of the inhibited vCA1 neuronal activity in nociceptive behaviors (Fig. 6).

In contrast, noxious stimuli excited more pyramidal neurons than innocuous stimuli under both physiological and pathological conditions. The excitatory response features were instrumental in the encoding and discrimination of stimulus properties even during neuropathic pain (Fig. 3). However, instead of reducing mechanical pain thresholds, chemogenetic activation of vCA1 pyramidal neurons had an analgesic effect on neuropathic pain (Fig. 7). We conjecture that only specific activation of the neurons with an excitatory response to noxious stimuli can lower the pain threshold [36], while chemogenetic activation mainly reverses the overall suppressed vCA1 pyramidal neuronal activity, thus alleviating mechanical allodynia. Here, a conflict that needs to be noted is that recent research reports that activation of the dorsal, but not the ventral hippocampus relieves neuropathic pain [37]. This discrepancy might be due to the difference in the activation scope and cell type in the hippocampus.

The functional heterogeneity of separate neuronal populations in one brain region is hardly new. This may be attributed to disparate upstream or local circuit modulation [38], divergent downstream innervation [39, 40], or distinct molecular expression in these populations [41, 42]. A significant fraction of the vCA1 putative interneurons exhibited an excitatory response to plantar stimuli, and comparison of the precise onset time of neural activity change in response to noxious laser stimulation revealed that the laser-evoked excitation of both pyramidal neurons and interneurons preceded the laser-evoked inhibition of pyramidal neurons (data not shown). Thus, we posit that the inhibited activity of vCA1 pyramidal neurons might be driven by local interneurons which provide general inhibition to pyramidal neuronal activity [43]. In particular, the somatostatin-expressing interneurons that show increased activity after peripheral inflammation are likely to be the potential inhibitory source for vCA1 pyramidal neurons. In addition, the entorhinal cortex and amygdala provide the main excitatory glutamatergic input of the hippocampus and are both excited by pain [7, 44]. Thus, we speculate that pyramidal neurons in vCA1 that exhibit excitatory responses to noxious stimuli may receive primarily excitatory inputs from these two afferent regions. Further studies to elucidate the upstream modulation of the stimulus-inhibited or -excited vCA1 neurons are warranted. Moreover, the CA1 area is the main efferent structure of the hippocampus [45], and it targets various pain-related brain regions like the medial prefrontal cortex [20], amygdala [46], and nucleus accumbens [47]. A previous study has shown that vCA1 projection neurons route distinct behavior-contingent information selectively to different target areas [40]. Yet whether the vCA1 pyramidal neurons with inhibitory or excitatory responses to peripheral sensory stimulation innervate divergent downstream regions remains an open question.

### The Role of vCA1 Theta Oscillation in Pain Modulation

Hippocampal theta has been associated with memory [48], anxiety [49], spatiotemporal encoding [50], and sensorimotor integration [51] among other emergent phenomena. It has been proposed that theta activation in the dCA1 parallels formalin-evoked nociception and noxious heat stimuli [10, 34]. Our results confirm similar findings in the vCA1, that enhanced theta power not only correlates with the persistent pain state but is closely related to instant nociceptive behaviors (Fig. 4). Although the pinprick stimulation evoked similar neuronal responses of vCA1 between the sham and SNI groups (Fig. 3), the firing of a remarkable fraction of vCA1 putative pyramidal neurons after stimulation was phase-locked to the local theta rhythm in the SNI group (Fig. 5), suggesting stronger processing and integration of nociceptive information in the vCA1 during neuropathic pain. We suppose that regulating the spiking activity in a specific theta phase, rather than recruiting more neurons to participate in the response, might be a more efficient manner of integrating nociceptive information.

The medial septum and diagonal band complex (MSDB) is critical for hippocampal theta generation [52]. The MSDB receives nociceptive information directly from the spinal dorsal horn [53, 54]. The septohippocampal neurons are activated following peripheral noxious stimulation [55]. It has been demonstrated that hippocampal pyramidal neurons are in general inhibited during theta activity, due to the excitation of local interneurons by the septal theta pacemaker cells [56]. Selective destruction of MSDB cholinergic neurons attenuates formalin-induced theta activation along with pyramidal neuron suppression, but not pyramidal neuron excitation in the dCA1 [57]. Given the well-established functional segregation along the longitudinal axis of the hippocampus that the dorsal part participates in episodic memories while the ventral part contributes to anxiety-like behaviors [14, 15], it remains intriguing to test whether similar procedures affect vCA1 theta and neuronal responses to nociception.

In addition, the hippocampal theta rhythm synchronizes many afferent and efferent structures [58]. Studies have shown that reduced dorsal and ventral CA1–prefrontal cortex theta connectivity is associated with impaired spatial memory and persistent spontaneous pain in chronic pain models, respectively [16, 20]. Increased ventral hippocampal–prefrontal theta synchrony correlates to anxiety, which is a comorbidity of chronic pain [32, 49]. The amygdala possesses nociceptive neurons that encode the unpleasantness of pain [7], and strong theta-frequency synchrony between the amygdala and ventral hippocampus has been recorded during the processing of aversive stimuli [59, 60]. This evidence underlines the prominent role of the hippocampus as an integration node in nociceptive information processing, which coordinates other pain-related structures through theta synchronization and participates in different dimensions of pain.

**Fig. 8:**
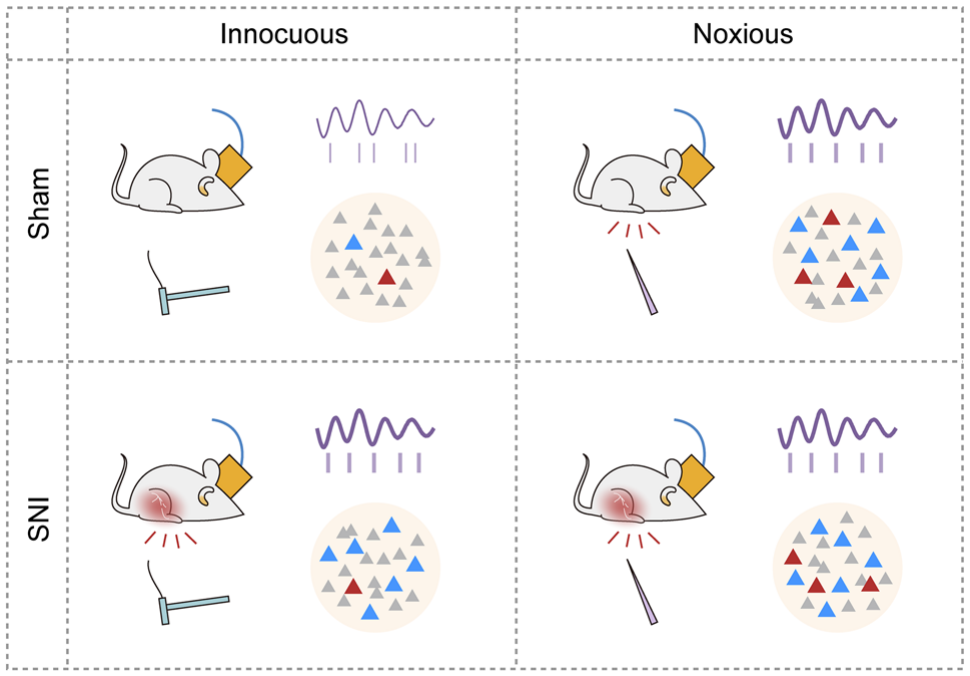
Schematic of the representations of nociception in the vCA1. Nociception is characterized in pyramidal neuron activity by a large fraction of suppression (blue triangles) and a small fraction of activation (red triangles), along with enhanced theta power and theta-spike synchronization (sine curve and raster in purple) in vCA1. The activity of inhibitory response neurons parallels the nociceptive behaviors and plays a causal role in the mechanical allodynia during neuropathic pain, while the activity of excitatory response neurons mirrors the actual stimulus intensity and is instrumental in the discrimination of stimulus properties.

In conclusion, our study provides direct evidence for the representation of nociceptive information in the vCA1 (Fig. 8). The functional heterogeneity of the nociception-inhibited and -excited neurons underlies a novel paradigm of neural coding of sensory stimuli. Taken together, these results speak for the critical role of the ventral hippocampus in pain perception and modulation.
